# Digital proficiency: assessing knowledge, attitudes, and skills in digital transformation, health literacy, and artificial intelligence among university nursing students

**DOI:** 10.1186/s12909-024-05482-3

**Published:** 2024-05-07

**Authors:** Ebtsam Aly Abou Hashish, Hend Alnajjar

**Affiliations:** 1 College of Nursing - Jeddah, King Saud bin Abdul-Aziz University for Health Sciences, Jeddah, Saudi Arabia; 2 King Abdullah International Medical Research Center, Jeddah, Saudi Arabia; 3 Faculty of Nursing, Alexandria University, Alexandria, Egypt

**Keywords:** Digital transformation, Digital health literacy, Artificial intelligence, Knowledge, Attitude, Skills, University nursing students, Perception, Technology

## Abstract

**Background:**

Implementing digital transformation and artificial intelligence (AI) in education and practice necessitates understanding nursing students’ attitudes and behaviors as end-users toward current and future digital and AI applications.

**Purpose:**

This study aimed to assess the perceived knowledge, attitudes, and skills of nursing students regarding digital transformation, as well as their digital health literacy (DHL) and attitudes toward AI. Furthermore, we investigated the potential correlations among these variables.

**Methods:**

A descriptive correlational design was employed in a Saudi nursing college utilizing a convenience sample of 266 nursing students. A structured questionnaire consisting of six sections was used, covering personal information, knowledge, skills and attitudes toward digital transformation, digital skills, DHL, and attitudes toward AI. Descriptive statistics and Pearson correlation were employed for data analysis.

**Results:**

Nursing students exhibited good knowledge of and positive attitudes toward digital transformation services. They possessed strong digital skills, and their DHL and positive attitude toward AI were commendable. Overall, the findings indicated significant positive correlations between knowledge of digital transformation services and all the digital variables measured (p = < 0.05). Senior students reported greater digital knowledge and a positive attitude toward AI.

**Conclusion:**

The study recommends an innovative undergraduate curriculum that integrates opportunities for hands-on experience with digital healthcare technologies to enhance their digital literacy and skills.

**Supplementary Information:**

The online version contains supplementary material available at 10.1186/s12909-024-05482-3.

## Background

The adoption and use of modern information and communication technologies (ICTs) have become integral to daily life [1]. Therefore, digital transformation has emerged as a significant revolution in many countries, including Saudi Arabia. The Saudi Vision 2030 and the National Transformation Program (NTP) were launched in 2015 to modernize healthcare systems through technology and innovation. To achieve this transformation, various components of health information technology, such as electronic health records, telemedicine, virtual consultations, online scheduling, and unified medical records, are being strengthened [2–4]. The global healthcare sector is also placing great importance on the adoption and utilization of health information technology, making it a top priority in healthcare transformation plans. To successfully implement and utilize these technologies, healthcare professionals and students need to be educated in health informatics and computer literacy [5].

## Conceptual framework

This study operates within a conceptual framework encompassing many digital variables, such as digital transformation knowledge, attitudes, skills, DHL, and attitudes toward AI among university students:

*The digital transformation* concept refers to the infusion of digital technology across all facets of a business or organization. This process reshapes business operations by leveraging digital tools to enhance efficiency, accessibility, and scalability [6]. Amidst of digital transformation, healthcare has evolved significantly, introducing innovations to improve health systems in response to rising global health needs, including infectious and chronic diseases [7]. The World Health Organization (WHO) outlines various digital tools, such as computers, mobile health (mHealth), software, telemonitoring, and artificial intelligence (AI) algorithms, spanning telemedicine, digital health games, and more [8]. Despite its potential, complete integration of digitalization into clinical practice remains a challenge. There is a gap between the potential effectiveness of digitalization and its actual implementation in healthcare [9].

Moreover, the digitalization of health care has changed the roles and responsibilities of healthcare professionals [10]. Likewise, students, as future health professionals, need to develop digital knowledge and skills with a positive attitude to match evolving needs [11]. Additionally, there is a need to identify the impact of and understand how to best use digital health [12]. The ability to use digital health refers to “*digital health literacy*” (DHL). The WHO has defined digital health (or eHealth) as the use of digital technologies in activities related to health. The DHL reflects the digital skills and ability to search for, understand, use, and evaluate health information on digital media (web-based and mobile), actively participate in health information exchange and interaction, and use the obtained information to address or solve health problems [13].

Digital health services can strengthen the healthcare system by improving the efficiency of care in hospital settings, enhancing adherence to clinical guidelines [14], providing opportunities to support clinical practice, and advancing patient-centric care [15]. Additionally, technology has the potential to improve access to information, enhance interactions between patients and healthcare providers, and lead to enhanced DHL [16]. High levels of digital health literacy were shown to be associated with better health outcomes for patients, nurses, and medical professionals, as well as for the public [17]. A lack of digital health literacy is the main barrier to people integrating into a digital society and enjoying convenient and efficient digital health services [18].

In addition, *artificial intelligence (AI)* is one of the trending applications of digital transformation and comprises many healthcare technologies that transform nurses’ roles and enhance patient care [19, 20].

Artificial intelligence (AI) refers to the application of computers and technology to replicate intelligent behavior and problem-solving akin to that of a human being [21]. Artificial intelligence (AI) has become indispensable in healthcare, aiming to enhance patient outcomes and streamline healthcare services. Through the utilization of machine learning algorithms, natural language processing, and computer vision, AI facilitates the analysis of intricate medical data. Its integration into healthcare systems seeks to aid clinicians, customize patient treatments, and improve overall population health, all while tackling the obstacles posed by escalating expenses and constrained resources [22]. Likewise, nursing AI tools encompass clinical decision support, mobile health, sensor-based technologies, voice assistants, and robotics. These devices have the potential to automate routine tasks, optimizing nurses’ time for direct patient care [23]. AI applications include analyzing electronic nursing records, pressure sores and safety risk analysis, nursing robots, and scheduling [24, 25].

Despite positive performance and utilization expectations from the application of AI technology in the healthcare setting, there are growing alarms. Using AI simultaneously increases the risk of unforeseen outcomes, discrimination, and ethical issues caused by malfunctions and incomplete technology related to AI medical devices; the alteration and bias of information due to a lack of accumulated data or learning errors in AI; and the invasion of privacy, which is also growing [26] and might be dangerous to the nursing profession [23]. However, Briganti and Le Moine (2020) emphasized the rising importance of AI in healthcare and recommended incorporating AI education into medical and nursing school curricula. Hence, investigating nursing students’ attitudes toward AI is crucial for ensuring their future preparedness [27].

### Significance of the study

Nurses and nursing students play a crucial role in introducing, implementing, and using technology in clinical practice, and possessing digital literacy and skills upon completing nursing baccalaureate studies is crucial [7]. Digital and AI literacy is increasingly used as a core requirement of students, academics, patients, and healthcare professionals due to the importance of technology in nursing education and practice [16]. Successful implementation of digital transformation applications and AI in clinical practice requires a thorough understanding of the attitudes and behaviors of nurses as end-users toward existing and future AI applications. Moreover, assessing the current knowledge of AI among nurses and nursing students is essential for identifying future training requirements, as they are technology users and have direct contact with patients [28].

Previous research studies have shown that the use of technology predominantly depends on one’s knowledge and attitude toward that technology, including AI [8, 23, 26–28]. While there is a consensus on the importance of digital literacy for nursing students, there is limited focus on measuring digital transformation and skills among Saudi nursing students. To the best of our knowledge, no study has investigated the knowledge, attitudes, and use of digital transformation, along with nurses’ relationships with digital literacy and attitudes toward AI. This study fills a gap by offering baseline assessment data for nurse educators and insights for enhancing nursing curricula. It is valuable for researchers, students, and academicians in the digital transformation era, offering guidance on developing educational programs to better prepare future healthcare professionals for the digital shift in healthcare.

### Aim of the study

The aim of this study was to explore the perceived knowledge, attitudes, and skills of nursing students regarding digital transformation, as well as their digital health literacy (DHL) and attitudes toward AI. Additionally, the study seeks to identify potential correlations among perceptions of digital transformation, DHL, and attitudes toward AI.

## Methods

### Study setting and design

This research employed a descriptive cross-sectional design at the College of Nursing-Jeddah (CON-J), which admits female students and is affiliated with King Saud bin Abdul-Aziz University for Health Sciences, National Guard Health Affairs, in Jeddah, Saudi Arabia.

### Subjects

The study included a convenience sample of undergraduate nursing students in the third and fourth academic years of 2022/2023, excluding preparatory year students. While the Raosoft Sample Size Calculator suggested a minimum sample size of 169 based on certain parameters (population size 300, margin error of 5, confidence interval 95%), invitations were extended to all eligible students to prevent missing data, resulting in responses from 266 students.

### Data collection instrument

A structured questionnaire composed of six sections was used to collect all the required data. The first four sections were developed by the current researchers based on a relevant literature review [1, 9, 15–23, 25, 26, 28–31, 40, 43, 46, 56–58].

Section 1: A personal information form containing questions inquiring about the students’ age, academic level, Internet use, and frequency of digital device use was used. Section 2: Knowledge of Digital Transformation: This knowledge section included 19 questions asking about digital transformation, examples, usability, ease of use, impact of digital technologies, and factors stimulating or hindering the use of digital technologies. Responses to these questions used multiple response options, such as “5” (useful /definitely yes) to “1” (not useful not familiar). Section 3: Attitudes toward Digital Transformation (ATD): This section included 11 items about students’ attitudes toward digital transformation and digital services. Section 4: Digital skills (DS): This section included six items where the respondents provided their opinions on their level of digital skills in the use of digital technologies, online public services, and taking advantage of digital learning opportunities. Section 5: Digital Health Literacy (DHL): The DHL scale consists of eight items adapted by current researchers from Mekawy et al. [23] to assess students’ perceived skills in digital health literacy. Section 6: Attitudes toward artificial intelligence (AAI): The AAI scale was adapted from Abuzaid et al. [28]. The scale includes five items about students’ attitudes toward artificial intelligence in nursing, which is one of the major digital transformations. Responses in Sects. 3 to 6 were framed on a 5-point Likert scale ranging from “5” strongly agree to “1” strongly disagree.

### Developed instrument sections, validity, and reliability

The developed parts of the study instrument (Sects. 1- 4), encompassing personal information, knowledge of digital transformation, attitudes toward digital transformation (ATD), and digital skills (DS), were meticulously proposed by the current researchers and underwent a rigorous development process as follows:

*Generating Items*: The process began with an extensive literature review conducted by the researchers, consulting diverse sources including academic journals and reputable websites to gather pertinent information on digital transformation in the healthcare sector. Utilizing databases such as PubMed, Google Scholar, and relevant websites facilitated access to scholarly articles and research studies pertaining to digital transformation. Employing various search strategies, including specific keywords like “digital transformation,” “digital transformation in healthcare,” “nurse education and digital skills,” and “attitudes towards technology in healthcare,” the researchers synthesized the gathered information. Subsequently, a list of items covering different aspects such as personal information, knowledge of digital transformation, attitudes towards digital transformation, and digital skills was generated based on the literature review and search. *Questionnaire Development*: Following the item generation phase, the researchers proceeded to develop the questionnaire in English and structured it into four sections. Emphasis was placed on ensuring clarity, relevance, and comprehensiveness of the questionnaire items. The wording of questions was reviewed and refined to enhance understandability and appropriateness for the target population of nursing students.

### Validity and reliability

The whole questionnaire underwent thorough evaluation for face and content validity. Five academic experts individually assessed its relevance, comprehensiveness, and clarity. They unanimously agreed that all items were easily understood, with only minor revisions required of some words. The content validity index (CVI) was calculated at 95.36, indicating high consensus among experts regarding item relevance. A pilot study involving 5% of nursing students further confirmed all questionnaire’s sections clarity and applicability. Based on feedback from experts and the pilot study, minor adjustments were made to the final version of the questionnaire. In addition, internal consistency was assessed using Cronbach’s alpha, yielding satisfactory values of 0.752, 0.726, 0.880, 0.782, and 0.742 for the digital transformation knowledge, ATD, DS, DHL, and AAI questionnaires, respectively.

### Data collection

After Institutional Review Board (IRB) approval was obtained, the researchers distributed the questionnaires to the nursing students who consented to participate in the study during their designated break times upon agreement. Detailed instructions were provided by the researchers, and on average, each student spent approximately 25 min completing the questionnaire. The data collection phase spanned two months within the final semester of the academic year 2022–2023.

### Data analysis

The data were statistically analyzed using the Statistical Package for the Social Sciences (SPSS) version 25. The demographic characteristics are presented as frequencies and percentages. Descriptive statistics, including the mean and standard deviation, were used to summarize the variables. Student’s t test and analysis of variance (ANOVA) were employed for means comparisons. Pearson correlation coefficient analysis (r) was used to explore the relationships among the digital variables. The predetermined significance level for *p* values was set at *p* ≤ 0.05.

### Ethical considerations

The study obtained IRB approval from CONJ and King Abdullah International Medical Research Center (KAIMRC) (IRB Approval: NRJ23J-034-01). Before participation, the researchers explained the study’s purpose, emphasizing participants’ rights to refuse or withdraw without academic consequences. Informed consent was obtained, ensuring data privacy and confidentiality.

## Results

### Participant characteristics

Successfully, 266 female students actively participated in this study; the mean age was 22.75 (SD. 2.59) years, and the majority of the students were older than 20 years. Among them, 51.1% were in their third academic year and did not take the informatics course, while the remaining 48.9% were in their fourth academic year and took the informatics course. A large proportion (85.3%) frequently utilized the internet, primarily through smartphones (84.6%) and tablets or iPads (78.9%). Refer to Table 1 for further details.

**Table 1:** Demographic data of the nursing students (*N* = 266)

Item	Total (*N* = 266)	
	No.	%
Age		
≤ 20–22	36	13.5
> 22–24	230	86.5
Mean (SD).	22.75(2.59)	
Academic year		
3rd	136	51.1
4th	130	48.9
Take nursing informatics course		
No	136	51.1
Yes	130	48.9
Internet use		
Frequently	227	85.3
Moderately	34	12.8
Rarely	5	1.9
Digital device frequently used #		
Smartphone	225	84.6
Tablet/IPad	210	78.9
Personal PC/Laptop	92	34.6

SD: Standard deviation #: Multiple answers

### Perceived digital transformation variables among nursing students

Table 2 shows that students had high overall knowledge of digital transformation services and applications (3.91 (0.55)). Students perceived high usability, ease of use, and good impact of digital technologies, as well as knowledge of the factors stimulating and hindering their use. In addition, students showed a moderate positive ATD (3.65 (0.46)), high overall DS (4.09 (0.74)) and DHL (3.72 (0.76)), and a moderate positive AAI (3.42 (0.54)). See Supplementary Table 1 for more information on all domains. Moreover, senior students in the fourth year compared with students in the third year had higher knowledge of digital transformation services and applications (t = 2.179, *p* = 0.030), DS (t = 2.126, *p* = 0.034), and DHL (t = 5.487, *p* = 0.001). However, there was no significant difference between them in terms of their ATD and AAI. See Table 2 for more values.

**Table 2:** Mean scores of the studied digital variables according to total and academic level

Perceived digital variables	Total (*N* = 266)	Academic level for students		t	*p*
		3rd year (*n* = 136)	4th year (*n* = 130)		
	Mean (SD)	Mean (SD)	Mean (SD)		
Overall knowledge of digital transformation	3.91(0.55)	3.86(0.51)	4.02(0.62)	2.179	0.030^*^
ATD	3.65(0.46)	3.61(0.43)	3.74(0.51)	1.966	0.051
Overall DS	4.09(0.74)	4.02(0.75)	4.23(0.71)	2.126	0.034^*^
Overall DHL	3.72(0.76)	3.55(0.70)	4.07(0.76)	5.487	< 0.001^*^
Overall AAI	3.42(0.54)	3.42(0.57)	3.43(0.48)	0.081	0.935

t: Student’s t test p: p *: statistically significant at *p* ≤ 0.05.

Mean values: 1–2.5 = low mean (< 50%); >2.5–3.75 = moderate mean(50–75%); >3.75–5 = high mean (75%).

### The highest perceived factors stimulating or hindering the use of digital technologies

Table 3 illustrates the highest perceived factors stimulating or hindering the use of digital technologies. Students identified a faster and more reliable internet connection (81.2%), a wider dissemination of digital tools (79.3%), and public awareness (75.6%) as the top stimulating factors. Conversely, approximately a quarter of the students (25.94%) highlighted insufficient user knowledge or skills; scarcity of budgets, facilities, and supportive resources (23.31%); and concerns about information security breaches and privacy invasion as the principal hindrances to the use of digital technologies.

**Table 3:** Perceived factors stimulating or hindering the use of digital technologies

Factors	No.	%
Stimulating Factors (*N* = 216)		
A faster and more reliable internet connection	216	81.2
Widespread of tools the improve trust and reputation of digital technology	211	79.3
More public awareness of services as online medical prescription	201	75.6
Hinders (*N* = 69)		
Inadequate knowledge/skills of the user	69	25.94
Lack of budget, facilities and, supporting resources	62	23.31
Fear of information security breach and privacy invasion issues	62	23.31

### Correlation between knowledge of digital transformation and studied digital variables

**Fig. 1:**
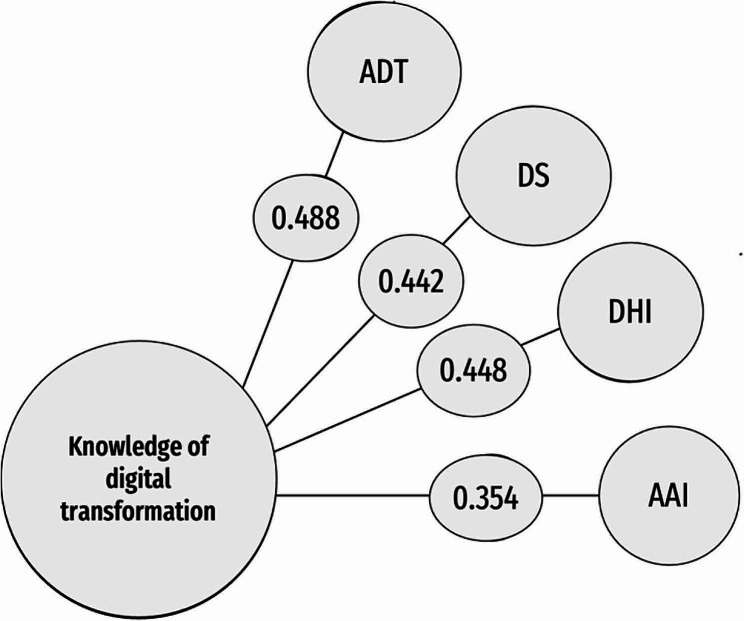
Correlation between knowledge of digital transformation and studied digital variables

Table 4; Fig. 1 show significant positive correlations between knowledge of digital transformation services and each of ATD, DS, DHL, and AAI (*r* = 0.488, 0.442, 0.448, 0.354, *p* < 0.001). See Table 4 for more values.

**Table 4:** Correlation between knowledge of digital transformation and studied digital variables

Variables		Knowledge of digital transformation	ATD	DS	DHL	AAI
Knowledge of digital transformation	r (p)	-	0.488 (< 0.001)^*^	0.442 (< 0.001)^*^	0.448 (< 0.001)^*^	0.354 (< 0.001)^*^
ATD	r (p)		-	0.699 (< 0.001)^*^	0.444 (< 0.001)^*^	0.531 (< 0.001)^*^
DS	r (p)			-	0.688 (< 0.001)^*^	0.559 (< 0.001)^*^
DHL	r (p)				-	0.235 (< 0.001)^*^

r = Pearson correlation, p significant

ATD: Attitudes toward digital transformation, DS: Digital Skills, DHL: Digital health literacy, AAI: Attitudes toward artificial intelligence

## Discussion

Currently, nursing students need robust digital knowledge and skills for effective healthcare in the technology-driven world. Our study explored various digital variables among nursing students, acknowledging the crucial role of technology in modern healthcare [29, 30]. The findings of the present study revealed that approximately half of the students studied informatics as an academic course, and the majority of the students frequently accessed the internet through their smartphones, tablets, or iPads. This aligns with the trend of informatics becoming a popular academic interest, reflecting technological advancements and students’ use of smart devices to facilitate internet access. This inclination toward technology usage could have potential positive impacts on both personal and academic aspects of their lives. Smartphones play essential roles in contemporary life, supporting information access, online engagement, social connections, entertainment, and academic learning. Their user-friendly design significantly contributes to increased reliance, notably among students.

This study aligns with previous research reporting the prevalent use of smartphone mobile technology devices for internet access among students, including Saudi students [31, 32], Egyptian nursing students [23], Nepalese nursing students [33], and Turkish nursing and medical students [34]. Students highly value internet access, particularly for health-related information, with smartphones as the primary means of internet usage. This reliance aligns with rapid lifestyle changes and blended learning integration in nursing education [23]. Previous studies have also highlighted the widespread adoption of mobile technology for educational purposes, including accessing course materials and leveraging online resources and collaborative exchanges between students and educators [35]. Students also engage in “edutainment” through social media. Al-Rahmi et al. advocate for educators to encourage social media applications to enrich the learning process [36]. However, Gabrovec et al. observed lower daily internet usage among Slovenian university students [37].

Regarding the perception of digital variables, the findings revealed that nursing students demonstrated good knowledge and awareness of digital transformation services, as these services are easy to use and have positive impacts on various aspects of such services. They also possess positive attitudes, good digital skills applicable in daily life and the job market, commendable digital health literacy, and positive attitudes toward artificial intelligence. These findings might reflect the readiness of students in the digital domain, which aligns with the pervasive transformation observed in various sectors and is consistent with the Saudi 2030 vision. These findings parallel those of many studies, such as Sheela [19], Harerimana and Mtshali [16], Mekawy et al. [23], and Keasberry et al. [14], which emphasize the impact of attitudes toward technology on individuals’ readiness to apply their knowledge and skills. Moderate to high digital health literacy was reported by Mekkawy et al. [23] and was linked to increased internet usage and nursing informatics integration, as well as a positive attitude toward AI application. Shanmugapriya et al. [38] and Leonardsen et al. [39] reported positive attitudes toward mobile technology among nursing students. These professionals strongly endorsed its role in expediting communication among healthcare professionals, improving patient care through information access, and recognizing the growing necessity of digital skills in the healthcare sector. Kwak et al. [40] reported that a positive attitude toward information technology reduces anxiety, promotes IT use, and enhances problem-solving confidence. In relation to AI, Abdelaliem et al. [31] found that nursing students exhibited a notably high level of enthusiasm for AI. However, the study suggested that this enthusiasm among nursing students might not be fully leveraged within their educational framework. Ronquillo et al. [25] indicated that nursing education still involves the integration of AI into curricula and lacks standardization in informatics competencies.

In contrast, challenges, such as low digital health literacy and digital knowledge impacting tool utilization, were highlighted in previous studies [9, 41]. Similarly, Kwak et al. [40] identified challenges stemming from limited digital knowledge, with more than 70% lacking a clear understanding of AI’s role. Moreover, Shudayfat et al. [42] reported that despite having daily Internet access and a positive attitude toward online health information, most students exhibited inadequate eHealth literacy scores. Rosenbaum et al. reported consistent identification of low health literacy levels, particularly in the domain of electronic health information [43].

Parallel with the descriptive level of the studied digital variables, the positive correlations found among the studied digital variables in this study revealed that students with a knowledge and awareness of digital transformation services and applications were more likely to hold positive attitudes toward them, exhibit higher digital skills and health literacy, and embrace attitudes toward artificial intelligence. This result highlights the strong link between digital transformation knowledge and adaptability to technological advancements, emphasizing its crucial role in informed decision-making and digital health practices. Positive attitudes play a pivotal role in technology acceptance in nursing education, as indicated by findings across multiple studies.

Farias-Gaytan et al. [44] accentuated the pivotal role of digital technology skills and knowledge for the current generation, with 95% of the study findings focusing on digital literacy and educational technology. This aligns with the emphasis on higher education institutions preparing students and educators with essential digital skills to meet the evolving demands of the workforce [44, 45]. Aavakare identified a correlation between digital literacy and the intention to use digital technologies, highlighting the impact of digital literacy on utilizing digital technologies for learning [46].

Researchers have also found that a positive attitude is linked to increased openness to digital applications, including AI, and is associated with reduced anxiety and enhanced problem-solving confidence among nursing students [23, 40]. Furthermore, Hunady et al. reported that positive expectations about the economy and life satisfaction from recent digital technologies correlate with favorable attitudes toward these technologies [47]. However, some studies reveal no significant correlation between AI scores and digital health literacy among nursing students, suggesting potential variations in educational backgrounds [23, 48]. The acceptance of AI applications by nursing students is notably influenced by their attitudes toward AI, with variations based on their level of digital device utilization [31]. In this respect, the significance of digital literacy, including AI literacy, in effective communication within nursing education and practice is emphasized by Spante et al. [49]. Chan et al. highlighted the improvement in digital literacy through training and perceived benefits, particularly in effective communication via digital platforms [50]. Additionally, Chen et al. (2018) emphasized the importance of acquiring digital health and AI literacy for academic success and future technology utilization upon completing a nursing degree [51].

Moreover, the study revealed that senior nursing students exhibited greater perceived knowledge, digital skills, and digital health literacy than juniors did. These findings align with previous studies that have highlighted the influence of academic level and increased clinical exposure on the development of career-related skills among nursing students [52, 53]. This result supports the idea that students will gain more experience as they progress to academic levels. This could be because they spend more time learning about nursing, which makes them more familiar with digital technologies and gives them more experience improving their digital skills. A variety of healthcare education resources, such as nursing informatics courses, may also be used. These findings align with previous research underscoring the influence of academic level on competency development, particularly given the advanced digital expertise of senior nursing students. Ibrahim and Aldawsari found a link between academic progress and self-confidence with the development of digital skills at the university level [30].

Additionally, Jeon and Kim (2022) underscored the positive influence of digital nursing education programs on academic performance, emphasizing the need for sufficient technological infrastructure [54]. In contrast, other studies have shown no significant demographic factors related to attitudes, knowledge, or skills related to DHL and AI [19, 23]. This variation among studies may stem from differences in study settings, technological advancements, and the degree of students’ reliance on digital life and digital tools.

Finally, the reported barriers and facilitators influencing the use of digital technologies by students align with findings from previous studies [9, 41, 55–57]. Gkrimpizi et al. identified more than 20 barriers to digital transformation in higher education institutions (HEIs), including challenges such as digital literacy, resistance to change, risk aversion, inadequate IT infrastructure, budget constraints, and privacy concerns [55]. Similarly, Borges do Nascimento et al. reported infrastructure issues, psychological barriers, personal concerns, and resource limitations [56]. Furthermore, Hunady et al. [47] noted insufficient IT infrastructure, safety concerns, and user skills, while Elsayed and Sleem and Alhasan et al. highlighted challenges in nursing education, particularly at the baccalaureate level, arising from a lack of basic digital knowledge, poor infrastructure, limited internet connectivity, and access to digital devices [9, 41]. Additionally, Dhirani et al. underscore information security and privacy issues as significant hurdles [57].

In contrast, addressing barriers in deploying digital health technologies through different strategies and facilitators is crucial. Borges do Nascimento et al. suggest that training programs, perceived technology effectiveness, and multistakeholder incentives facilitate adoption [56]. Songkram et al. identified key facilitators influencing students’ attitudes toward digital learning platforms, emphasizing factors such as perceived usefulness, ease of use, and technology self-efficacy [58]. Dhirani et al. recommended that ethical concerns be resolved before these advancements are deployed [56]. Gkrimpizi et al. emphasized the need for targeted measures from university authorities for successful digital transformation in HEIs. The authors recommended validating identified barriers through further research or stakeholder validation for accuracy and relevance [55].

### Strengths and limitations

This descriptive correlational study is important because it is the first to measure perceptions of various digital variables in a Saudi nursing college, providing robust insights into digital transformation. However, limitations exist, including the reliance on a single college sample, potentially limiting the generalizability of the findings to diverse nursing student populations in Saudi Arabia and beyond. Additionally, self-reported data introduce the possibility of response bias. While a positive correlation between knowledge and attitudes toward digital transformation was uncovered, a direct cause-and-effect relationship was not established. Factors such as personal experiences, organizational digital culture, and individual perceptions may also influence knowledge and attitudes. These limitations suggest avenues for future research exploration as in the recommendation section.

## Conclusion

The incorporation of digital transformation technology and AI in nursing education and practice is increasingly essential. This finding matches the findings of the present study, which revealed that nursing students demonstrated a robust understanding and positive attitudes toward digital transformation services and applications. They perceived these technologies as user friendly and acknowledged their positive impact. Nursing students showcased strong digital skills applicable in daily life and the labor market. Moreover, their commendable digital health literacy and positive attitude toward AI highlighted their overall preparedness in the digital realm. Significantly, positive correlations were observed between knowledge of digital transformation services and all the measured digital variables, with senior students exhibiting greater digital knowledge and a more positive attitude toward AI. These results emphasize the critical need to engage students with digital healthcare technologies to enhance their digital competency, ensuring their readiness for the evolving healthcare landscape and their future careers.

### Recommendations

*The present study suggested many fruitful implications for education, practice and future research.*

#### Education

Innovative approaches in nursing education are crucial for equipping students with essential digital competencies. One recommendation involves ensuring the provision of an undergraduate curriculum that integrates digital healthcare technologies such as informatics and AI. This step ensures that students gain hands-on experience, enhancing their digital competency. Training courses focusing on internet usage skills will further augment the digital capabilities of nursing students.

#### Practice

In nursing practice, the emphasis should be on diversifying the skill sets of nursing students. Encouraging them to acquire a wider range of skills, including digital literacy, genomics, AI, and machine learning, during their clinical training will enable them to adapt to the changing healthcare environment. By fostering continuous learning strategies, nurses can stay abreast of technological advancements and apply them effectively in clinical practice.

#### Research

Investigating how digital knowledge impacts decision-making, patient outcomes, and healthcare practices will contribute to the knowledge base and inform future strategies in healthcare education and practice. Further studies replicating this methodology could enhance the reliability and validity of the findings, especially considering the single-site nature of the study. Future research should involve a broader spectrum of nursing students across various academic years to explore the impact of education duration and technology exposure on digital literacy, knowledge, skills, and AI perceptions. Additionally, investigating the adaptation of university curricula to align with the transformations of NTP, emphasizing digital skill integration, is crucial.

### Electronic supplementary material

Below is the link to the electronic supplementary material.

Supplementary Material 1

## Data Availability

Data is provided within the manuscript or supplementary information files.

## Abbreviations

AI: Artificial intelligence
AAI: Attitudes toward artificial intelligence
ANOVA: Analysis of variance
ATD: Attitudes toward digital transformation
DS: Digital Skills
DHL: Digital health literacy
CI: confidence interval
CON-J: College of Nursing-Jeddah
IRB: Institutional Review Board
ICTs: Information and Communication Technologies
KAIMRC: King Abdullah International Medical Research Center
KSAU-HS: King Saud bin Abdulaziz University for Health Sciences
NTP: National Transformation Program
SPSS: Statistical Package of Social Sciences
WHO: World Health Organization
