# Supplementary material for: Digital proficiency: assessing knowledge, attitudes, and skills in digital transformation, health literacy, and artificial intelligence among university nursing students
Source: BMC Med Educ. 2024 May 7;24:508. doi: 10.1186/s12909-024-05482-3 (PMC11077799; doi:10.1186/s12909-024-05482-3)
Supplement: Supplementary file 1 — Supplementary Material 1 [file 12909_2024_5482_MOESM1_ESM.docx]

**Supplementary Table 1. Mean score of the studied variables according to total and academic level**

| **Perceived digital variables** | **Total**  **(N = 266)** | **Academic level for students** | | **t** | **p** |
| --- | --- | --- | --- | --- | --- |
|  |  | **3 year**  **(n = 136)** | **4 year**  **(n = 130)** |  |  |
|  | **Mean**± **SD** | **Mean**± **SD** | **Mean**± **SD** |  |  |
| **Overall perceived Knowledge** | **3.91 ± 0.55** | **3.86 ± 0.51** | **4.02 ± 0.62** | **2.179** | **0.030^*^** |
| 1- Knowledge of digital transformation services/ applications | 3.89 ± 0.84 | 3.84 ± 0.82 | 3.99 ± 0.86 | 1.313 | 0.190 |
| 2- Usability of digital transformation services/ applications | 4.05 ± 1.0 | 3.99 ± 0.96 | 4.16 ± 1.08 | 1.322 | 0.187 |
| 3- Ease of use of digital transformation services/ applications | 3.99 ± 0.87 | 3.87 ± 0.85 | 4.23 ± 0.85 | 3.228 | 0.001^*^ |
| 4- Impact of digital technologies | 3.82 ± 0.66 | 3.82 ± 0.64 | 3.83 ±0.69 | 0.154 | 0.878 |
| 5- Factors stimulating the use of digital technologies | 3.95 ± 0.72 | 3.87 ± 0.69 | 4.12 ± 0.75 | 2.658 | 0.008^*^ |
| **Attitudes toward digital transformation and digital services** | **3.65 ± 0.46** | **3.61 ± 0.43** | **3.74 ± 0.51** | **1.966** | **0.051** |
| **Perceived Digital Skills** |  |  |  |  |  |
| a-Everyday use of digital skills | 3.98 ± 0.90 | 3.87 ± 0.94 | 4.20 ± 0.78 | 2.759 | 0.006^*^ |
| b-Digital skills and labor market | 4.15 ± 0.75 | 4.10 ± 0.77 | 4.25 ± 0.72 | 1.494 | 0.136 |
| **Overall Digital Skills** | **4.09 ± 0.74** | **4.02 ± 0.75** | **4.23 ± 0.71** | **2.126** | **0.034^*^** |
| **Overall Digital health literacy** | **3.72 ± 0.76** | **3.55 ± 0.70** | **4.07 ± 0.76** | **5.487** | **<0.001^*^** |
| **Overall Attitudes toward artificial intelligence** | **3.42 ± 0.54** | **3.42 ± 0.57** | **3.43 ± 0.48** | **0.081** | **0.935** |

SD: Standard deviation t: Student t-test p: p *: Statistically significant at p ≤ 0.05

ATD: Attitudes toward digital transformation, DS: Digital Skills, DHL: Digital health literacy, AAI: Attitudes toward artificial intelligence
